# Improved Triacylglycerol Production in *Acinetobacter baylyi *ADP1 by Metabolic Engineering

**DOI:** 10.1186/1475-2859-10-36

**Published:** 2011-05-18

**Authors:** Suvi Santala, Elena Efimova, Virpi Kivinen, Antti Larjo, Tommi Aho, Matti Karp, Ville Santala

**Affiliations:** 1 Department of Chemistry and Bioengineering, Tampere University of Technology, Korkeakoulunkatu 8, Tampere, Finland; 2 Department of Signal Processing, Tampere University of Technology, Tampere, Finland

## Abstract

**Background:**

Triacylglycerols are used in various purposes including food applications, cosmetics, oleochemicals and biofuels. Currently the main sources for triacylglycerol are vegetable oils, and microbial triacylglycerol has been suggested as an alternative for these. Due to the low production rates and yields of microbial processes, the role of metabolic engineering has become more significant. As a robust model organism for genetic and metabolic studies, and for the natural capability to produce triacylglycerol, *Acinetobacter baylyi *ADP1 serves as an excellent organism for modelling the effects of metabolic engineering for energy molecule biosynthesis.

**Results:**

Beneficial gene deletions regarding triacylglycerol production were screened by computational means exploiting the metabolic model of ADP1. Four deletions, *acr1*, *poxB, dgkA*, and a triacylglycerol lipase were chosen to be studied experimentally both separately and concurrently by constructing a knock-out strain (MT) with three of the deletions. Improvements in triacylglycerol production were observed: the strain MT produced 5.6 fold more triacylglycerol (mg/g cell dry weight) compared to the wild type strain, and the proportion of triacylglycerol in total lipids was increased by 8-fold.

**Conclusions:**

*In silico *predictions of beneficial gene deletions were verified experimentally. The chosen single and multiple gene deletions affected beneficially the natural triacylglycerol metabolism of *A. baylyi *ADP1. This study demonstrates the importance of single gene deletions in triacylglycerol metabolism, and proposes *Acinetobacter *sp. ADP1 as a model system for bioenergetic studies regarding metabolic engineering.

## Background

Triacylglycerols are the main components in vegetable oils. TAGs are used for various purposes including food applications, cosmetics, oleochemicals and biofuels. Current raw materials for biodiesel and renewable diesel include vegetable oils, animal fats or recycled greases. The production potential of current vegetable oil sources is limited to replace commodity products derived from fossil resources. One alternative to produce TAGs is to utilize heterotrophic organisms which produce lipids from organic molecules, such as sugars. In addition to crop sugars, different waste and residue streams can be used as a source of sugars for heterotrophic TAG production. In the field of bacterial lipid production, significant yields of TAG have been obtained with *Rhodococcus opacus *PD630 which naturally stores large amounts of TAG, up to 76% of cellular dry matter [1,2].

In order to improve economic feasibility of the process the production strains need to be engineered and optimized [3]. Advances in synthetic biology and molecular engineering have enabled the tuning of metabolic pathways to increase the production yields. Deleting genes of competitive pathways and inserting novel activities can significantly improve the yield of a desired product. Steen *et al. *[4] published a study in which *Escherichia coli *was genetically engineered to produce fatty esters, fatty alcohols and wax esters straight from plant biomass containing simple sugars. Furthermore, Kalscheuer *et al. *[5] have launched the production of 'Microdiesel' in metabolically engineered *E. coli*. In this approach, lipid and ethanol formation was combined with subsequent esterification of fatty acids and ethanol, resulting in formation of fatty acid ethyl esters (FAEE). More recently, a pilot-scale process with FAEE producing *E. coli *was carried out [6].

*Acinetobacter baylyi *is a strictly aerobic and widely spread soil bacterium with simple growth requirements and wide substrate range. The laboratory strain ADP1 has shown potential for metabolic studies and biotechnological applications, mainly for the compact easily-transformable genome and a unique metabolic network [7]. The genome of ADP1 has been sequenced, revealing several similarities to the genome of *E. coli*, thus providing possibility to exploit the knowledge applied to this microorganism so far [8]. The genome sequence of ADP1 has provided bioinformatic backbone for construction of a constraint-based metabolic model and a single gene knock-out mutant library [9,10]. Furthermore, the strain is known to naturally accumulate storage lipids, including triacylglycerol (TAG). Lipid metabolism of *Acinetobacter *strains have been already studied to some extent and especially the bifunctional enzyme WS/DGAT have drawn a lot of interest [11-14]. Combining *in silico *(computational) and *in vitro *(experimental) techniques, the production rates can be potentially improved by target-specific gene knock-outs. Thus, the strain ADP1 serves as an excellent model organism for TAG production and metabolic engineering.

The objective of this work was to study the lipid metabolism of ADP1 and construct genetic tools for metabolic engineering. Potential single gene deletions affecting TAG metabolism were scanned *in silico *with the help of a genome-wide metabolic model and gene annotation data [10]. The effects of chosen gene deletions on TAG production were experimentally studied.

## Results

### *In silico *predictions for gene deletions

Flux balance analysis based method was used for identifying the single gene deletions that are potentially beneficial for TAG production. The deficiency of genes leading to improvement of lipid production can be due to blocking or silencing competitive metabolic reactions or pathways, activation of lipid production synthesis route, blocking or silencing lipid degrading pathway, or redirecting the lipid production towards different lipid groups. As a result of *in silico *calculations, literature, and predictions based on gene annotation data, a list of beneficial single gene deletions was obtained (Table 1).

**Table 1:** Gene deletions beneficial for TAG production found by *in silico *calculations exploiting the metabolic model of *A.baylyi *ADP1 or by predictions based on gene annotation.

gene ID	strain ID	Gene name	Product	EC	Spotted by (i = *in silico*, a = annotation)
ACIAD2837	M4	*dgkA*	diacylglycerol kinase	EC 2.7.1.107	i
ACIAD3383	M1	*acr1*	fatty acyl-CoA reductase	EC 1.2.1.n2	i,a
ACIAD2844		*glpD*	glycerol-3-phosphate dehydrogenase	EC 1.1.5.3	i
ACIAD2425		*cyoA*	cytochrome o ubiquinol oxidase subunit II	EC 1.10.3.-	i
ACIAD2426		*cyoB*	cytochrome o ubiquinol oxidase subunit II	EC 1.10.3.-	i
ACIAD2291		*cydB*	cytochrome d terminal oxidase polypeptide subunit II	EC 1.10.3.-	i
ACIAD3381	M2	*poxB*	pyruvate dehydrogenase (cytochrome)	EC 1.2.2.2	i
ACIAD3648		*estA*	carboxylesterase	EC 3.1.1.1	i,a
ACIAD1134		*aesT*	esterase	-	i,a
ACIAD3309	M3	*-*	lipase	EC 3.1.1.3	a
ACIAD1121		*lip1*	lipase	EC:3.1.1.3	i,a

### Assessment and verification of *in silico *predictions

**Figure 1:**
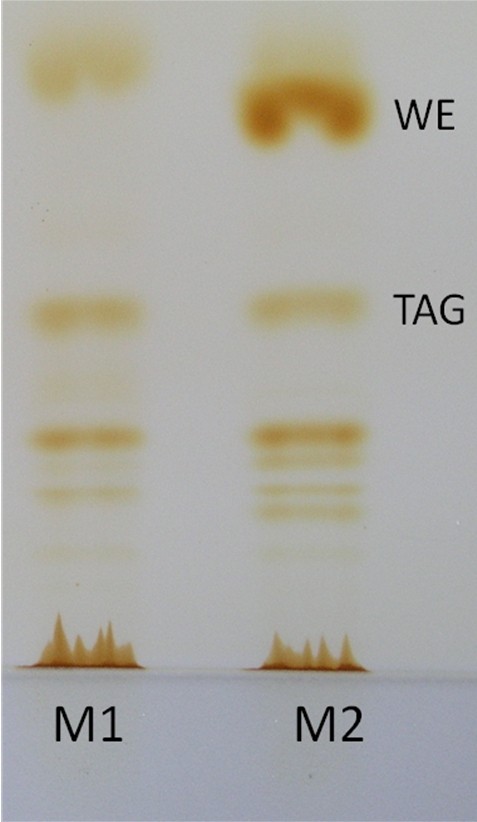
**Phenotyping of mutant strains M1 and M2 lipid fractions**. The strains M1 and M2 were cultivated in 50 ml MA/9 supplemented with 5% glucose and 0.2% cas.amino acids at 37°C and 300 rpm for 30 hours. Lipid extraction and thin layer chromatography were carried out as described in the text. The strain M1 was confirmed to be deficient of wax ester synthesis in contrast to M2.

According to the evaluation of the *in silico *model, gene annotation, and literature survey, four candidates representing very different mechanisms affecting the TAG pathway were chosen to be studied experimentally. The gene ACIAD3309 is annotated as a TAG lipase degrading the product of interest. A strain lacking a fatty-acyl-CoA reductase ACIAD3383 (*acr1*) is incapable of fatty alcohol biosynthesis and therefore wax ester synthesis, which is the end-product of a competitive storage lipid pathway. ACIAD3381 (*poxB*) is associated to acetate production, and therefore the deletion redirects the carbon flux towards storage lipid synthesis. The fourth deletion, ACIAD2837 diacylglycerol kinase (*dgkA*), catalyses phospholipid synthesis consuming 1,2-diacylglycerol, an important precursor of TAG, as a substrate. In preliminary tests, the phenotypes and growth characteristics of the mutant strains were determined. Few observations were made: it was verified by thin layer chromatography (TLC) analysis, that the strain ACIAD3383 (designated as M1) is unable to produce wax esters (Figure 1). For the strain ΔACIAD3381 (M2), the carbon flux was found to be shifted towards wax ester synthesis (Figure 1). For the strain ΔACIAD3309 (M3) drastic changes in phenotype or growth were not observed. Deletion of ACIAD2837 (strain M4) affected the cell growth negatively by increasing lag-time and decreasing the amount of biomass.

### Strain construction and characterization of TAG production

**Figure 2:**
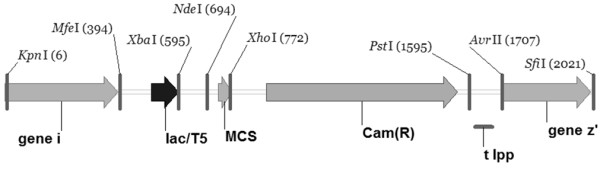
**Gene cassette**. The constructed gene cassette was used for creating triple gene deletion in the strain M3. Abbreviations of the components are explained in the text. Restriction enzyme recognition sites used for the construction are shown in the figure.

**Figure 3:**
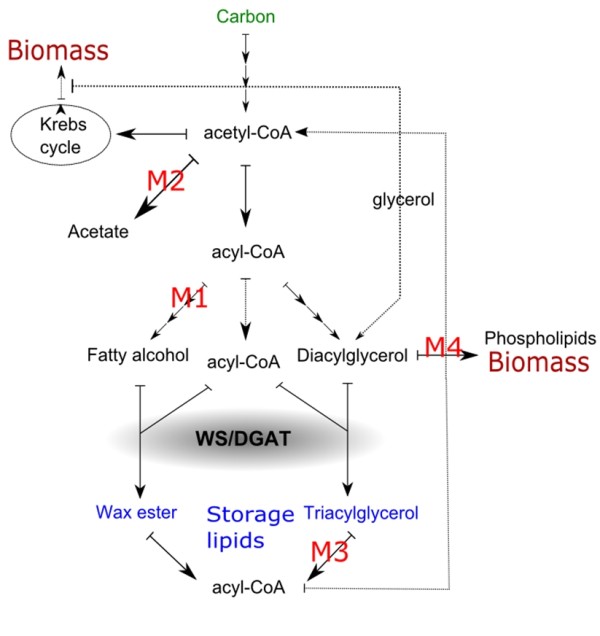
**The schematic representation of the lipid synthesis pathway of *A. baylyi *ADP1**. The carbon fate in ADP1 cells is distributed to biomass (including phospholipids), carbon dioxide and storage compounds, such as wax esters and triacylglycerols. The gene deletions of the studied strains M1 (ACIAD3383, Δ*acr1*), M2 (ACIAD3381, Δ*poxB*), M3 (ACIAD3309), and M4 (ACIAD2837, Δ*dgkA*), hypothetically affect the pathway as mapped in the figure. The main substrates of WS/DGAT are shown in the figure.

In order to study the cumulative effects of gene deletions on TAG production, a knock-out strain with three gene deletions was constructed (ACIAD3309, ACIAD3381 and ACIAD3383). The deletion ACIAD2837 was left out from the knock-out combination for the negative effect on growth properties. For strain construction, synthetic gene cassette was used (Figure 2). The gene cassette was constructed *in vitro *using well-characterized biocomponents: promoter (lac/T5), multiple cloning site (MCS), transcription termination loop (t lpp), selection marker (cam(r)), and homologous sequences from ADP1 (downstream of ACIAD3383 and upstream of ACIAD3381) to knock out selected target genes. The strain M3 was transformed with the gene cassette and selected on LA plate containing 50 μg/ml chloramphenicol. The strain was designated as MT. In addition to the target genes, the gene ACIAD3382 was deleted for practical reasons; the group of the three adjacent genes ACIAD3381 - ACIAD3383 could be deleted using single knock-out cassette. The gene ACIAD3382 encodes a homocysteine synthase, *metY*. The function is essential for methionine biosynthesis, but there is another gene present (ACIAD2314) in the genome possessing the same function, for which the deletion of the gene was not expected not to have an effect on the phenotype. The lipid synthesis pathway of ADP1 is presented in Figure 3.

TAG production of the wild type strain ADP1 and the knock-out strains M1, M2, M3, M4 and MT was experimentally studied in the laboratory. The six strains were cultivated in two-phase batch cultures in MA/9 minimal medium supplemented with sodium gluconate and glycerol as carbon and energy sources. The amount of biomass of different strains was determined, resulting in cell dry weights (CDW) 2.4 - 3.6 g/l with M4 possessing the lowest number and the strain M3 the highest.

**Figure 4:**
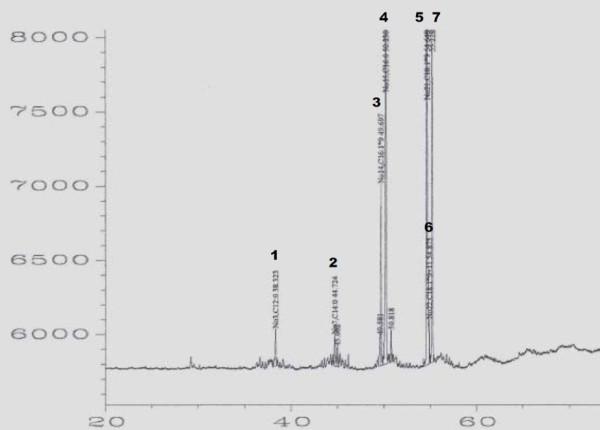
**Lipid profile of acylglyceride fraction of ADP1**. The lipid profile of acylglyceride fraction was analyzed with gas chromatography. In the figure: 1 lauric acid (C12:0), 2 myristic acid (C14:0), 3 palmitoleic acid (C16:1), 4 palmitic acid (C16:0), 5 oleic acid (C18:1n9c), 6 trans-9-&-cis-11-octadecenoic acid (C18:1n9+11), 7 stearic acid (C18:0).

**Figure 5:**
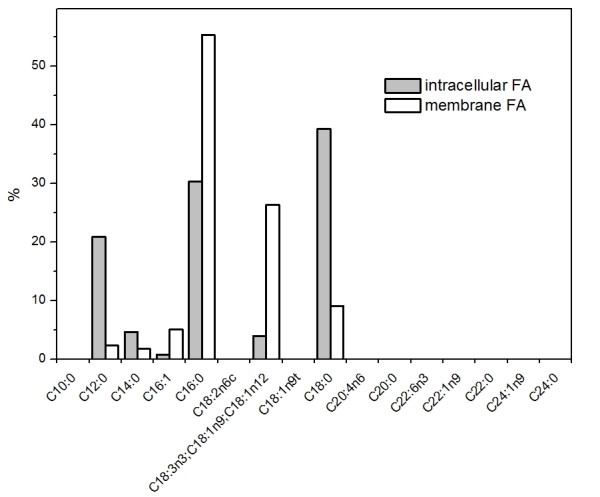
**Lipid profiles of intracellular and membrane lipid fractions of ADP1**. The intracellular and membrane lipids were analyzed with gas chromatography to determine the proportions of different fatty acids in the fractions. In the figure: FA, fatty acids.

The profiles of acylglyceride lipid fraction (Figure 4), and intracellular and membrane lipids (Figure 5) were analyzed by gas chromatography. The cytoplasmic lipids included C16:0, C18:0 and C12:0 as main fatty acids and C13:1, C16:1 and C14:0 fatty acids as minor constituents. In acylglyceride fraction (mono-, di-, triglycerides), C16 and C18 fatty acids were dominant. The profiles were found to be similar in all studied strains (data not shown).

For extracted lipids the amount of total lipids was determined gravimetrically. For visualization and quantitative analysis of specifically TAG, preparative thin layer chromatography (TLC) was carried out. The amount of total lipids (mg/g CDW) was found to be similar in all strains (Table 2). On the contrary, differences in relative TAG quantity were observed: the TAG content was higher in all studied deletion strains compared to the wild type strain and the strain MT produced 5.6 fold more triacylglycerol (mg/g CDW) than the wild type strain. Moreover, the proportion of TAG in total lipids increased significantly (Table 2). As the *in silico *model predicted, the lipid synthesis was shifted towards the intended lipid fraction. The most drastic changes were observed for the MT strain (8 fold improvement in TAG per total lipids), indicating that combining the deletions have cumulative effects on TAG production.

**Table 2:** Biomass and lipid yields of the strains.

Strain	CDW (g/l)	Total lipids (mg/g CDW)	TAG/total lipids
wild type	3.25 ± 0.04	77 ± 6	1.6%
M4	2.43 ± 0.03	87 ± 4	ND
M2	3.24 ± 0.02	78 ± 9	ND
M3	3.56 ± 0.02	67 ± 5	7.6%
M1	2.85 ± 0.07	60 ± 3	7.8%
MT	2.68 ± 0.17	54 ± 2	12.4%

ND not detected, CDW cell dry weight, TAG triacylglycerol.

The utilization of gluconate and glycerol, and the production of end metabolites were determined by High Performance Liquid Chromatography (HPLC). The presence of common end-products such as acetate, lactate and succinate was studied in ADP1 cultivations. Detectable amounts of the above mentioned known metabolites were not seen in any of the samples, indicating that carbon is mainly directed to CO_2_, biomass, and storage compound production. Also, no significant differences were observed in carbon usage between the knock-out strains; within the first 24 hours of cultivation dedicated as biomass accumulation conditions (phase I), approximately 20-35 mM of gluconate was consumed. Within the next 24 hours (phase II, growth conditions favouring storage compound production), gluconate consumption decreased to approximately 13-25 mM. According to optical density measurements, after transferring the cells to low nitrogen medium (phase II) the amount of biomass did not increase significantly. Thus, the consumed carbon was directed to other functions than biomass formation, as intended. The consumption of glycerol did not correlate with the measured TAG content of the cells, indicating that the endogenous glycerol synthesis does not limit the TAG production in the studied conditions. Moreover, for all the strains, glycerol was only consumed within the first 24 hours and only to minor extent (1-10 mM).

## Discussion

Modelling the biochemical pathways and studying the effects of competitive or beneficial key enzymes in specific routes increase the knowledge and understanding of microbial metabolism [15]. Metabolic engineering has shown importance also in developing sustainable and feasible biological processes for microbial lipid production. In order to investigate the possibilities to increase the TAG accumulation of *Acinetobacter baylyi *ADP1 by target specific gene knock-outs, the lipid production of the strain was studied. Potentially beneficial gene deletions were screened by computational means exploiting the constraint-based metabolic model available for ADP1. The whole genome sequence database enabled the construction of genetic tools for specific molecular engineering to take place *in vivo*.

According to *in silico *results and literature, the four most interesting gene deletions with different action mechanisms on TAG synthesis pathway were chosen to be studied more in detail both individually and concurrently. To study the cumulative effects of gene deletions, three of the deletions were combined to form a strain with four gene deletions (three target genes and one additional gene). As the goal was to shift the lipid metabolism towards TAG synthesis, the fatty acyl-CoA reductase ACIAD3383 (*acr1*) was deleted, totally blocking the wax ester (WE) synthesis [16]. The cells are incapable of converting long chain acyl-CoA to the corresponding fatty aldehydes which are intermediate products of WE production. Due to the lack of WEs, the amount of total lipids was found to be lower than in the wild type strain but the amount and proportion of TAG increased, indicating that WE production competes with TAG production to some extent. Furthermore, the gene deletion seemed not to have a negative effect on cell growth and biomass formation.

The gene ACIAD3309 is annotated as a TAG lipase, and the corresponding knock-out strain M3 was experimentally shown to possess good cellular fitness and slightly increased TAG accumulation in the studied conditions. However, the role of the lipase in context of intracellular lipid metabolism is unclear, since the gene sequence contains clear signal sequence, indicating subcellular location and potential involvement in periplasmic or membrane lipid metabolism. Thus, further studies are required to show the interplay of different lipases in storage lipid metabolism.

In *E. coli*, the gene *poxB *(corresponding to ACIAD3381) is associated to acetate production and carbon flux [17,18]. In *E. coli*, acetate accumulation inhibits growth and bioprocess control systems are required to neglect the negative effects of acetate [19]. The deletion is interesting in ADP1 since the strain was not recognized as being prone to acetate formation. On the other hand, acetate plays a role in various biochemical pathways intracellularly, which cannot be detected by end-metabolite analyses. However, detectable amounts of acetate or other end-products are not formed in any of the strains and therefore the link between *poxB *deletion and carbon fate is not straightforward. The deletion did not shift the carbon metabolism towards TAG production in M2 strain but rather towards WE formation in studied conditions, as shown in Figure 1. Interestingly, according to some studies the WE formation has been also associated to over-flow metabolism in hexadecane grown cells [20]. Thus, one of the hypothesis of using the *poxB *deletion for the wax ester deficient MT strain was that the carbon flow could be shifted from WE production towards TAG production. Since there is only one enzyme, WS/DGAT, responsible for the final step of producing both WE and TAG, such modulation in theory is possible.

The strain MT with four gene deletions (ACIAD3309, ACIAD3381, ACIAD3382, ACIAD3383) was constructed using a synthetic gene cassette. For practical reasons (see: strain construction) ACIAD3382 was also deleted, although the gene is not known to be involved in lipid metabolism. The TAG content of the cells relatively increased compared to the single gene knock-out mutants and the wild type strain. Also, the lipid metabolism was shifted towards producing higher percentage of TAG of total lipids. On the other hand, the amount of total lipids and biomass decreased slightly. Without supplementation of convenient substrates or over-expression of the key enzymes of the route, it is difficult to obtain the dual goal of high cell density and high cellular lipid content, since the biomass and lipid production are competing for the same building blocks. Furthermore, as the TAG production pathway was not engineered as a continuum, it is possible that the potentially increased substrate flux for WS/DGAT was insufficient, due to either a naturally low expression level of WS/DGAT [12] or the re-direction of the substrate away from TAG production. However, the strain MT was found to be the most TAG producing strain among the studied strains and in that sense the computational evaluation of the target gene deletions was successful. Also, the computational results were calculated under the steady-state assumption while the experimental results are from batch experiments.

Supplementation of the medium with glycerol did not seem to have effect on TAG accumulation. According to analyses, glycerol had been only consumed within the first culture phase. This indicates that glycerol is consumed rather as a carbon and energy source in biomass formation than building up TAG. The lack of glycerol consumption in phase II can be related to the induction of the endogenous glycerol synthesis as a TAG backbone or just simply to the fact that cells in stationary phase do not import glycerol to the cells.

In a bioenergy application point of view, few observations can be done. The results of qualitative lipid analyses show that the main constituents of the intracellular lipids are C16 and C18 fatty acids, with minor proportions of C12 and C14. The C18 and C16 fatty acids are generally the main components in vegetable oils, such as rapeseed oil, soy bean oil or palm oil, and desirable raw materials for biodiesel or renewable diesel [21], suggesting the suitability of the ADP1 TAGs to be exploited in fuel applications. It is also known that the proportion of saturated lipids can be increased by increasing the cultivation temperature [22]. Furthermore, the substrate utilization of ADP1 was found to be straightforward, as demonstrated by HPLC analyses. This suggests that suitable pathways for re-directing the carbon flux towards desired products can potentially be found, since the number of different routes is limited. These features are valuable in bioprocesses, as undesired end-metabolites are not formed, and process control is simple. However, the natural production rate of TAG in ADP1 is rather low. Here, the proportion of TAG in total lipids was improved by using simple gene knock-outs that re-direct the carbon flow towards TAG production but overall yield of TAG still remained low. Despite the several favorable features of ADP1 regarding carbon usage and bioprocess overall, the process needs to be further optimized before exploiting the strain as a full-fledged production host.

Using a computational analysis to screen beneficial gene deletions from a genome-wide metabolic network allows going through a number of deletions that would be impossible manually. Also, capturing effects of upstream deletions on the product yields can be difficult without modelling. However, rapid and sensitive high through-put assays are not yet available for analyzing large number of samples, although some suggestions for alternative methods have been made [23]. In addition to lack of straightforward execution method for predicted gene deletions, modelling lipid metabolism alone is challenging for many reasons; the production and accumulation of storage lipids is known to be under strict regulation, and TAG production mostly takes place in the stationary growth phase, limiting the applicability of growth-coupled analysis methods (such as OptKnock). The model applied in this study does not account for any changes in regulatory mechanisms, which can strongly affect reaction rates. At the moment such phenomena cannot be modelled, but in theory it is possible by e.g. changing either the intake reaction rates or objective function in flux balance analysis calculations as a function of time and environmental state. Making such modifications to the model might improve predictions and explain why potentially significant deletions were missed. On the other hand, by experimental observations the model can be improved. For effective bidirectional enhancement of model and process realization, a large number of different gene knock-outs and their combination should be evaluated to reveal the potential bottle necks and limitations of a bioprocess design. To obtain sufficient improvements and to build up an economical bioprocess, profound understanding of the cellular groundwork in lipid metabolism is required. This study demonstrates how single or multiple gene deletions of the natural pathway present detectable changes in TAG production, providing new aspects in over-production of valuable biomolecules.

## Conclusions

In this article, we demonstrated the significance of understanding the changes in natural lipid metabolism in response to simple gene knock-outs. The predictions of the constraint-based metabolic model regarding beneficial gene deletions for TAG production were experimentally verified. *Acinetobacter baylyi *ADP1 proved to be a potential platform for a microbial cell factory and a model system for studies involving metabolic engineering. The strain naturally produces TAG, and the lipid profile corresponds to that in vegetable oils and can potentially be used to replace or as a supplement to vegetable oil for various purposes, including biofuel applications. The TAG production of *A. baylyi *ADP1 was demonstrated to improve by genetic knock-outs.

## Methods

### *In silico*

Computational modelling and simulation was performed using a genome-wide reconstruction of *A. baylyi *metabolism [10]. The reconstruction was extended by adding transport and exchange reactions for triacylglycerol (TAG) which enabled to simulate the accumulation of TAG. In order to identify deletions that potentially increase TAG production, we aimed at blocking pathways competing for the same precursor metabolites with TAG formation. This was implemented as follows:

(1) The main TAG producing pathway was identified, starting from glycerol-3-phosphate and acyl-CoA.

(2) The first reactions from each pathway diverging from the TAG producing pathway were selected. The set of marker reactions *R *was composed of reactions consuming glycerol-3-phosphate and acyl-CoA for fatty acid, fatty aldehyde, and fatty alcohol production. Additionally, the reactions consuming TAG or TAG precursors of variable carbon chain lengths, such as 1,2-diacylglycerol, were considered.

(3) Gene deletions were simulated by enforcing the respective enzyme-catalyzed reaction rates to zero.

(4) The maximal achievable rates of the marker reactions in *R *were calculated using flux balance analysis [24].

(5) If the studied gene deletions lowered the maximal achievable rate of a marker reaction considerably (the difference between the maximal rate of the marker reaction in the deletion and wild-type strain was greater than 0.15), the deletions were considered potentially beneficial for TAG production.

After running the algorithm the maximal TAG production was simulated for the identified gene deletions to verify that the TAG production itself was not blocked. *In silico *gene knockouts and flux balance analysis were carried out using COBRA toolbox [25] and GNU Linear Programming Kit http://www.gnu.org/software/glpk/). The analysis considered only knock-outs of genes that have been found to be non-essential to *A. baylyi *[9]. The modeled medium was similar to the minimal salts medium used in the wet lab experiments containing the key components required for growth and TAG production, including O_2_, Na^+^, H^+^, SO_4_^2-^, Fe^2+^, NH_4_^+^, PO_4_^- ^and a carbon source. The analysis results were not dependent on the carbon source.

### Strains

*Acinetobacter baylyi *ADP1 was used as the wild type strain (available at German Collection of Microorganisms and Cell Cultures, under accession number DSM 24193). The single gene knock-out strains M1, M2, M3, and M4 (gene deletions ACIAD3383, ACIAD3381, ACIAD3309, and ACIAD2837, respectively) were kindly provided by Veronique de Berardinis (Genoscope, France). In the single gene knock-out mutants, the gene in question is replaced with a gene cassette containing a kanamycin resistance gene (*kan^r^*) [9].

### Genetic engineering

The molecular work was carried out by using methods described by Sambrook *et al. *[26]. For digestions and ligations, the enzymes and buffers were provided by Fermentas (Lithuania) and used according to provider's instructions. PCR reagents were provided by Finnzymes (Finland) (DNA polymerase Phusion™ and buffer) and Fermentas (nucleotides). Primers were ordered from ThermoFisher Scientific (USA) with appropriate restriction sites, and the annealing temperatures were calculated according to Finnzymes' instructions. The primer sequences and descriptions are listed in Table 3.

**Table 3:** List of primers used in this study.

Name	Description	Oligo Sequence (5→3')
ab3	lac/T5, sense, *Mfe*I	TATACAATTGGCTGGCATCCCTAACATATCC
ab4	lac/T5, antisense, *Xba*I	ATATTCTAGAAATTCAAATTGTTATCCGCTCAC
ab5	MCS, sense, *Xba*I	CCCCTCTAGAAATAATTTTGTTTAACTTTAAGAA GGAG
ab7	kan(r), sense, *Xho*I	ATATCTCGAGTAACTAACTAACAATAAAACTGTCTGCTTACATAAACAG
ab8	kan(r), antisense, *Pst*I	TATACTGCAGTTAGAAAAACTCATCGAGCATCA AATG
ab9	t lpp, sense, *Pst*I	ATATCTGCAGGCTTGACCTGTGAAGTGAAAAAT G
ab10	t lpp, antisense, *Avr*II	TATACCTAGGGCTTAATGCGCCGCTACAG
ab49	cam (r), sense, *Xho*I	AATTCTCGAGATATGTATCCGCTCATGTCGAG
ab50	cam (r), antisense, *Pst*I	AATTCTGCAGCGTTTAAGGGCACCAATAACTG
ab55	Gene Z' (ACIAD3383), sense, *AvrII*	AAGCCCTAGGTATTCCCAAGCTCAACGGCTGC
ab56	Gene Z' (ACIAD3383), antisense, *Sfi*I	CCTCGGCCCCCGAGGCCCAGGTGACTGCTATGA ATGTTCACTATAATATTC
ab57	Gene i (ACIAD3381), sense,*Kpn*I	ATATGGTACCCACACCAATTTTAGCACCCGGAAAAAATG
ab58	Gene i (ACIAD3381), antisense, *Mfe*I	CCAGCCAATTGGTCGGGTCGAGTAGATGACGTT AAAAATTTG

The transformation of ADP1 was carried out by methodology described by Metzgar *et al. *[7]. Briefly, a linear DNA fragment (PCR product) with flanking regions of the target site in genome was inserted to ADP1 cultivation in an exponential growth phase. The cultivation was conducted at 30°C and stirring of 300 rpm using LB medium supplied with 1% glucose. For transformation, 1-2 μg DNA was used per 1 ml of cultivation. After insertion, the cultivation was incubated for 2-3 h and then spread on a selective LA plate supplemented with chloramphenicol (50 μg/ml) and glucose. The plate was incubated at 30°C until colonies appeared. Negative controls were cultivated in the same method except for insertions sterile water was used instead of DNA fragments. The construct in the obtained strain was verified with colony PCR and further by sequencing.

#### In vitro construction of a gene cassette for multiple gene knock-outs

The six gene cassette components were amplified separately by PCR: flanking region Gene_I (upstream of ACIAD3381) was amplified from ADP1 by colony PCR with primers ab57 and ab58 and flanking region Gene_Z'(downstream of ACIAD3383) with primers ab55 and ab56, respectively. The promoter T5 (lac/T5) was amplified from plasmid pCSS810 [27] with primers ab3 and ab4. Initially, the selection marker *kan(r) *was used, amplified from the plasmid pET-28 (Novagen, USA) with primers ab7 and ab8 and cloned back to the plasmid *in vitro *using restriction enzymes *XhoI *and *PstI *and T4-DNA-ligase. The resulting plasmid was used as a PCR template for amplifying multiple cloning site (MCS) and *kan(r) *together with primers ab5 and ab8. Later on, *cam(r) *(primers ab49, ab50, from pAK400c [28]) was used as a selection marker, and cloned to the gene cassette using restriction sites *XhoI *and *PstI*. Transcription termination loop (t lpp) was amplified from plasmid pAK400c with primers ab9 and ab10. Double digestions were carried out for the PCR products with restriction enzymes, and ligated in pairs. The ligation reactions were amplified by PCR with corresponding primers, digested again, and two of the pairs were ligated and amplified by PCR again. The two- and four-gene component sets were ligated and the whole gene cassette construct was amplified by PCR with primers ab57 and ab56, the final product being 2025 bp long. Purification of the PCR products was carried out in every step using PCR purification kit (Fermentas) or gel extraction kit (Fermentas). PCR products were run on 1-2% agarose (Sigma, USA) gel supplied with SYBRsafe (Invitrogen, USA) and visualized with SafeImager (Invitrogen).

### Medium composition

The following medium composition was used for the TAG cultivation of *A. baylyi *strains: Na2HPO_4 _· 2 H_2_O 5.518 g/l, KH_2_PO_4 _3.402 g/l, NH4Cl 1 g/l (phase I) or 0.1 g/l (phase II), nitrilotriacetic acid 0.008 g/l, NaCl 1.0 g/l, FeCl_3 _0.487 mg/l, FeSO_4 _· 7 H_2_O 5.6 mg/l, MgSO_4 _· 7 H_2_O 250 mg/l, CaCl_2 _· 2 H_2_O 20 mg/l, NaCl 117 mg/l, MnSO_4 _· 4 H_2_O 0.56 mg/l, ZnSO_4 _· 7 H_2_O 0.140 mg/l, Co(NO_3_)_2 _· 6 H_2_O 0.150 mg/l, CuSO_4 _· 5 H_2_O 0.130 mg/l, Na_2_MoO_4 _· 2 H_2_O 0.120 mg/l, H_3_BO_4 _0.160 mg/l, EDTA III 22.7 mg/l. Casein amino acids were added at concentration (0.2 w-%) for the phase I cultivation. Sodium gluconate (0.11 M) and glycerol (0.05 M) were used as a carbon and energy source.

### Cultivation

The batch cultivation was carried out in 100 ml medium/250 ml Erlenmeyer flasks. In the phase I, the strains were cultivated for 24 h in normal MA/9 medium at 37°C and 300 rpm. For phase II, the cells were collected by centrifugation (30 min., 3000 g) and suspended to fresh medium with reduced nitrogen concentration (0.1 g/l NH_4_Cl), in order to promote TAG accumulation [29]. The cultivation was continued additional 24 h in same conditions. After the cultivation, the cells were collected by centrifugation (45 min, 3000 g), freeze-dried, and the biomass of the samples was determined gravimetrically.

### End-metabolite analysis

End-products of the cultivations were measured with high performance liquid chromatography (HPLC). Culture samples were centrifuged at 20000 g for 5 minutes. Supernatant was collected and filtered through polycarbonate filter (Chromafil^® ^PET -45/25, Macherey-Nagel, Germany). The samples were analyzed for gluconate, acetate, succinate, fumarate, ethanol and lactate with LC-20AC prominence liquid chromatograph (Shimadzu, USA) equipped with RID-10A refractive index detector, DGU-20A5 prominence degasser, CBM-20A prominence communications bus module, and SIL-20AC prominence autosampler. Shodex SUGAR SH1011 (Showa Denko KK, Japan) kept at 40°C was used as a column. Sulfuric acid (0.01 N) was used as an eluent at pumping rate of 0.6 ml/min. Identification and quantification of liquid end products was based on co-chromatography of using standards. Mediums were used as controls.

### Lipid extraction

The lipids were extracted from the freeze-dried biomass samples using the Bligh and Dyer method [30] with two parallel samples. A total of 40 ml of original culture containing 70 - 135 mg of freeze-dried cells was extracted with 5 ml of chloroform, 10 ml of methanol and 4 ml of PBS buffer (ratio 1:2:0.8 v/v/v). The cell suspension was mixed well and shaken for 2 h at 150 - 200 rpm. The mixture of 5 ml of chloroform and 5 ml of PBS buffer (1:1 v/v) was added, suspension was mixed and stored overnight in a refrigerator at + 4°C. The suspension was centrifuged at 7000 g for 10 min. The lower (chloroform) phase was collected into a pre-weighed glass vial and evaporated under nitrogen. The extraction was repeated by adding 10 ml of chloroform to the upper water-methanol phase containing the cells and after mixing incubated for 40 hours at + 4°C. Finally, the phase separation was completed by centrifugation at 7000 g for 20 min. The chloroform phase was transferred to the glass vial with the first extract and purged under nitrogen. The amount of total lipids was determined gravimetrically.

### TLC analyses

In order to visualize the total lipid composition of the strains M1 and M2, TLC analyses were carried out using 10 × 10 cm HPTLC Silica Gel 60 F_254 _glass plates with 2.5 × 10 cm concentrating zone (Merck, USA) and dyed with iodine for visualization. Of extracted lipids, 10 μl of a sample was applied on the TLC plate. Mobile phase used was n-hexane:diethyl ether:acetic acid 90:15:1. The preparative chromatography for the isolation of the specific fraction of TAG from the total lipid extracts was carried out on Silica Gel 60 F_254 _10 × 20 cm glass plates with the concentrating zone 2.5 × 10 cm (Merck). The concentrated solution of the total lipid extract in chloroform (5 - 8 mg of total lipids in 50 μl of chloroform) was applied as a single band to a Silica Gel plate. Tripalmityol-glycerol (Sigma) or olive oil was used as a standard. The TLC chromatogram was developed with the solvent system n-hexane:diethyl ether:acetic acid 80:20:2. Iodine was used for visualization. After evaporation of iodine TAG-zone was scraped and transferred into a Pasteur pipet containing cotton wool. TAG fraction was eluted from Silica Gel with chloroform (3 × 0.7 ml). Chloroform was purged under nitrogen and the amount of TAG was determined gravimetrically.

### GC analyses

#### Fractionation of lipids by SPE

Fractionation of ADP1 wild type lipids was conducted using a custom-made solid-phase column. The column was prepared by filling Florisil^®^-SiO_2 _(Merck, 60 - 100 mesh size, heated at 120°C) into a glass pipette. The dried lipids were dissolved in 200 μl of chloroform. The silica column was washed with 3 ml of chloroform and n-hexane after which the lipid solution was transferred to the column. Three ml of n-hexane was added to the column and allowed to drain by gravity. The collected lipid fraction containing acylglycerides was purged with gentle nitrogen stream.

#### Intracellular and membrane lipid identification

In order to separate and identify intracellular and membrane lipids, sonication was used for cell disruption. Four ml of PBS buffer was mixed with a cell pellet and the sonication was conducted 3 times for 5 min (Soniprep 150, MSI, England). After the treatment, the sample was centrifuged at 10000 g for 10 min. The supernatant (intracellular fraction) was dissolved in 5 ml of chloroform and 10 ml of methanol. The remaining pellet (membrane fraction) was dissolved in 5 ml of chloroform, 10 ml of methanol, and 4 ml of PBS buffer.

#### FAME derivation

For determination of fatty acid profiles of acylglyceride fraction and to compare the fatty acid profiles the ones of intracellular and membrane lipids, the fatty acids were transesterified to form fatty acid methyl esters (FAME). The lipids were suspended in 200 μl toluene and 100 μl BF_3 _· MeOH (Sigma) and heated up for one hour at 100°C. One ml of distilled water and 1 ml of n-hexane were added and the reaction mixture was shaken for 2 minutes and centrifuged at 2000 g for 5 minutes. The upper n-hexane phase was collected into a new vial and evaporated under nitrogen. The n-hexane extraction and evaporation steps were repeated. The FAME sample was dissolved in dichloromethane and transferred into a GC glass vial. The sample was washed once by evaporating the dichloromethane and dissolving it again.

#### GC run

The fatty acid methyl esters were analyzed with HP-GC5890 gas chromatograph equipped with flame ionization detectors (FIDs). Injection volume was 1 μl for all samples. A fused silica column HP-5MS (5% phenyl/95% methyl silicone, 30 m × 0.25 mm × 0.25 μm film) was used with nitrogen as a carrier gas. Hydrogen and air were used as support gases. Injector and detector temperatures were both set to constant 250°. The run was carried out using following temperature program: 50°C for 10 min, followed by 4.18°C/min for 55 min, followed by 20 min at 280°C. Bacterial Acid Methyl Ester (BAME) mix (Supelco^®^, Sigma) with carbon chains C10:0 - C20:0, C22:0, C24:0, C26:0 and derivatives of the chains C15 - C19 was used as a standard.

## Competing interests

The authors declare that they have no competing interests.

## Authors' contributions

SS and VS designed the study. SS performed the molecular work, microbiological work, is responsible for the qualitative lipid analyses, and wrote the manuscript. EE carried out the quantitative lipid analyses and participated in manuscript drafting. VK, AL and TA are equally responsible for designing and performing the *in silico *work and took part in drafting the manuscript. VS and MK supervised and coordinated the study. All authors read and approved the final manuscript.
